# Affiliated Workforce Ageing and Shifting Injury Incidence Rates of the Ecuadorian Construction Sector

**DOI:** 10.1002/puh2.70454

**Published:** 2026-11-13

**Authors:** Antonio Ramón Gómez‐García, Cesar Eduardo Espinoza Samaniego

**Affiliations:** ^1^ Universidad Espíritu Santo Samborondón Ecuador

**Keywords:** aging, construction industry, developing countries, employment, occupational injuries

## Abstract

**Introduction:**

The construction sector in developing economies is facing a dual challenge: an ageing workforce and evolving occupational safety risks.

**Objective:**

To analyze an evolution of temporary disability occupational injuries (TDOIs) incidence rates (IR) among affiliated workers in the Ecuadorian construction sector, segmented by age groups.

**Methods:**

A longitudinal study (2010–2024) was conducted using official administrative records from the General Insurance for Work Risks (Seguro General de Riesgos del Trabajo [SGRT]). Age‐specific IRs per 1000 workers were calculated. Time‐trend analysis was performed using simple linear regression, Durbin–Watson (DW) tests for residual independence, and log‐linear generalized linear models (GLM) to estimate annual percentage changes (EAPC) in injury IRs across cohorts.

**Results:**

The study identified a significant inversion of risk within the sector. The youngest cohort (≤24 years) experienced a marked decrease in injury IRs, falling from 16.6 to 3.7 per 1000 workers (*p* < 0.001). In contrast, the oldest group (≥45 years) was the only cohort to show a significant increase, with IRs rising from 2.8 to 6.9 (*p* < 0.001). By 2024, workers aged ≥45 years reached the highest IR (6.94 per 1000 workers; 95% confidence interval [CI]: 6.05–7.98), significantly surpassing the youngest group (3.70 per 1000 workers; 95% CI: 2.46–5.57) and displaying a positive long‐term trend (EAPC = +4.20%; *p* < 0.001).

**Conclusion:**

The traditional paradigm of the young worker as the primary risk group has shifted. There is an urgent need for age‐specific prevention policies according to the needs of the ageing affiliated workforce in Ecuador.

## Graphical abstract

This study reveals a demographic shift in Ecuador's construction sector from 2010 to 2024, showing an ageing affiliated workforce alongside declining overall injury rates. However, workers aged ≥35 years exhibited a rising proportion of injuries, highlighting the need for age‐targeted occupational safety interventions and tailored health policies.

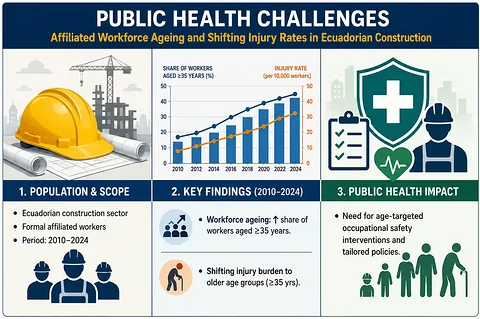

## 1 Introduction

Globally, the construction sector remains one of the most hazardous industries, bearing a disproportionate burden of work‐related injuries, illnesses, and fatalities [1]. This elevated risk of injury stems from physically demanding tasks, unpredictable job‐site conditions, and frequent exposure to numerous hazards. Falls from height are a leading cause of both fatal and nonfatal injuries in construction [2]. Fatigue from long working hours and insufficient rest is common, further increasing injury risk and reducing safety performance [3]. The complexity and variability of construction work often expose workers to multiple overlapping hazards, underscoring the need for strong safety management and effective mitigation strategies [4]. Despite some reduction in injury rates over the past three decades, the construction industry still records a high incidence of serious work‐related injuries, highlighting the ongoing need to improve occupational safety and health standards [5].

Within this context, age is a factor influencing both the likelihood and severity of occupational injuries in construction. Younger workers face at least twice the risk of injury compared to older workers, often due to inexperience, limited safety training, and a greater likelihood of engaging in high‐risk tasks [6]. In contrast, older workers are more likely to suffer severe injuries when accidents occur, leading to longer recovery periods, extended absences from work, and higher medical and compensation costs [7, 8].

However, this traditional epidemiological pattern may be shifting due to a reduced entry of younger workers into the industry and broader demographic changes that are driving the workforce age structure toward older cohorts [9, 10]. Consequently, workforce aging has emerged as a primary occupational safety and health challenge, underscoring the need for adaptive and age‐sensitive prevention strategies tailored to the specific needs of older workers [11].

Developed economies have implemented robust regulatory frameworks and age‐management strategies to reduce occupational risks for older workers. Key measures include workplace health promotion [10], ergonomic interventions [12], and targeted safety programs designed to support an aging workforce [13], which contribute to longer and safer career trajectories [14]. In contrast, developing countries in Latin America are undergoing rapid demographic changes while continuing to rely on outdated protection systems. Therefore, occupational safety and health policies in these regions remain age‐blind and have not kept pace with the aging workforce [15], significantly limiting the effectiveness of regional frameworks [16]. As a result, older workers face increased risks of work‐related injuries due to the absence of effective, age‐sensitive policies [17]. Addressing this challenge requires urgent reforms and the development of prevention strategies tailored to the needs of an aging workforce.

In the Republic of Ecuador (hereinafter, Ecuador), the construction sector constitutes a strategic pillar for the national economy, contributing between 6% and 8% of the gross domestic product [18]. Furthermore, it acts as a primary catalyst for the labor market [19], despite exhibiting an informal employment rate of 70% and a marked male predominance [20]. In parallel, national studies based on administrative records from the General Insurance for Work Risks (Seguro General de Riesgos del Trabajo, SGRT) identify construction as the sector with the highest rate of occupational injuries [21], followed closely by the manufacturing sector [22]. Notably, this high incidence persists despite the recent implementation of technical regulations aimed at addressing sector‐specific risks.

Currently, the dynamics of occupational injuries in relation to the shifting age structure of the Ecuadorian construction sector remain unexplored. Therefore, the objective of this study is to analyze longitudinal trends in age‐specific occupational injury incidence rates (IRs) within the Ecuadorian construction sector and to identify potential patterns of risk convergence in the context of a progressively aging workforce. We hypothesize that the progressive demographic aging of the affiliated construction workforce is linked to a significant shift in the occupational safety risk profile, with injury IRs declining among younger cohorts and rising among older workers, ultimately resulting in a risk inversion.

## 2 Materials and Methods

### 2.1 Study Design and Data Sources

A descriptive, longitudinal time‐trend study was conducted for the period 2010–2024, using a secondary analysis of administrative records from the Ecuadorian Social Security Institute (Instituto Ecuatoriano de Seguridad Social, IESS). The study population consisted exclusively of salaried construction workers classified under ISIC/Ecuador Code F who were formally affiliated with the SGRT [23]. The analysis focused on the annual frequency of officially recognized temporary disability occupational injuries (TDOIs) [24]. All data used in this study are publicly available and aggregated. In accordance with international ethical standards for secondary data research, formal ethics committee approval was not required, as the study did not involve identifiable personal information. This approach is consistent with both the International Ethical Guidelines for Health‐related Research Involving Humans [25] and the Organic Law on the Protection of Personal Data of Ecuador [26].

### 2.2 Age Group Classification

On the basis of occupational health criteria and the availability of the structure of the available administrative records, the study population was stratified into four age groups to represent different stages of working life and aging: young workers (≤24 years), early‐career workers (25–34 years), mid‐career workers (35–44 years), and older workers (≥45 years). This classification aligns with commonly used epidemiological methods for studying workforce ageing [27]. It enables a clear distinction between early‐career vulnerability, frequently driven by brief job tenure and incomplete safety training, and the cumulative physiological changes associated with later career stages in physically demanding sectors such as construction [28].

### 2.3 Statistical Analysis

Data were processed using Microsoft Excel (v. 16.78) and analyzed with Jamovi (v. 2.3.21.0). The statistical analysis was conducted in three sequential stages. First, a descriptive analysis was conducted to calculate the annual percentage distributions of affiliated workers and TDOIs, stratified by age group. This stage aimed to identify demographic shifts and evaluate the relative distribution of injuries across cohorts over the study period (2010–2024). To complement these descriptive metrics, exploratory simple linear trend models (*Y* = *β*_0_ +* β*_1_ *X *+ *ε*) were fitted to these proportions. Regression assumptions were formally evaluated: First‐order serial autocorrelation was tested using the Durbin–Watson (DW) statistic; heteroscedasticity and residual normality were examined via residual diagnostics; and the coefficient of determination (*R* ^2^) was reported to assess overall model fit. These linear models were strictly limited to evaluating exploratory proportional trends, whereas formal inferential modeling of injury rates was addressed in the third analytical stage.

Second, annual age‐specific IRs (operationally defined as annual cumulative injury risk per 1000 affiliated salaried construction workers) were calculated by dividing the annual frequency of TDOIs by the total affiliated workforce within each age group [24, 29]. This normalization enabled the comparison of injury risk across cohorts by accounting for fluctuations in the number of insured individuals over time [29]. Corresponding 95% confidence intervals (95% CI) for annual IRs were estimated assuming a Poisson distribution, based on the standard error of the natural logarithm of the rates (SE[ln(rate)] = 1/√(cases)).

Third, jamovi software module used for the estimation (GAMLj) with a Poisson log‐link distribution were fitted to quantify long‐term temporal trends in IRs across age groups. To model rates directly rather than raw counts, the natural logarithm of the annual affiliated workforce within each age group was included as an offset variable (ln(workforce)). In the presence of overdispersion (deviance/df > 1.2), a negative binomial log‐link GLM was applied. The estimated annual percentage change (EAPC) and its corresponding 95% CIs were derived from the unstandardized log‐linear slope coefficients (*β*_1_) using the exponential transformation formula: EAPC = 100 × (exp(*β*_1_) − 1). The EAPC derived from these log‐linear models represents the overall average long‐term annual percentage change across the full 15‐year study period (2010–2024), serving as a robust summary metric of macro‐level temporal trajectories rather than assuming a strictly constant annual rate of change throughout the entire series. To formally test the central hypothesis of differential temporal trends across cohorts, a unified negative binomial generalized linear model (GLM) was fitted incorporating Year (as a continuous variable), Age Group (categorical, with ≤24 years as the reference group), and a Year × Age Group interaction term, using the natural logarithm of the annual affiliated workforce as an offset (ln(workforce)). The statistical significance of the interaction term directly evaluated whether slope trajectories differed significantly across age cohorts: log(*E*[*Y*]) = *β*_0_ + *β*_1_Year + *β*_2_AgeGroup + *β*_3_ (Year × AgeGroup) + log(Workforce).

Furthermore, to address potential non‐stationarity, heteroscedasticity, and structural breaks introduced by the COVID‐19 pandemic, a formal sensitivity analysis was conducted by re‐estimating all generalized linear regression models on a restricted dataset excluding the peak pandemic period (2020–2021). Parameter stability and EAPC consistency between the complete series (2010–2024) and the restricted series (2010–2019 and 2022–2024) were formally evaluated. All statistical tests were two‐tailed, and statistical significance was established at *p* < 0.05.

## 3 Results

### 3.1 Demographic Distribution and Exploratory Trend Analysis of the Workforce

The affiliated SGRT construction workforce in Ecuador experienced a marked demographic shift between 2010 and 2024 (Table 1; Figure 1). Exploratory linear trend models explained a substantial proportion of temporal variance across all age groups (*R* ^2^ = 0.714–0.931; *p* < 0.001) and confirmed significant first‐order serial autocorrelation in each model (DW = 0.243–1.097; *p* < 0.05). The proportion of workers aged ≤24 years declined from 17.11% to 7.35%, and those aged 25–34 years decreased from 32.54% to 27.48% (Figure 1A). In contrast, the proportion of workers aged 35–44 years increased from 23.40% to 31.05%, whereas those aged ≥45 years rose from 26.96% to 34.12% (Figure 1B).

**TABLE 1:** Annual percentage distribution of affiliated construction workers in Ecuador by age group and exploratory linear trend analysis (2010–2024).

Year	Age group				Total, *N*
	≤24 (%)	25–34 (%)	35–44 (%)	≥45 (%)	
2010	17.11	32.54	23.40	26.96	64,553
2011	18.83	32.32	22.73	26.11	91,009
2012	18.77	32.22	22.81	26.20	118,620
2013	18.61	32.44	23.12	25.83	143,873
2014	17.54	32.53	23.68	26.25	149,658
2015	17.01	32.57	24.09	26.33	150,381
2016	14.55	32.33	24.89	28.23	118,222
2017	13.68	32.41	25.37	28.54	116,390
2018	12.16	32.02	26.06	29.77	109,002
2019	11.02	31.69	26.65	30.64	103,493
2020	8.56	31.11	27.95	32.38	78,363
2021	6.97	30.41	27.77	34.84	86,037
2022	8.00	29.66	30.29	32.04	88,520
2023	8.78	28.71	29.79	32.72	91,401
2024	7.35	27.48	31.05	34.12	84,416
Intercept (*β*_0_)	1960.676	638.840	−1205.906	−1292.962	
Slope (*β*_1_)	−0.965	−0.301	0.611	0.656	—
(95% CI)	(−1.138, −0.761)	(−0.415, −0.186)	(0.413, 0.809)	(0.473, 0.839)	—
*p* value^a^	*<0.001*	*<0.001*	*<0.001*	*<0.001*	—
Model fit (*R* ^2^)	0.901	0.714	0.931	0.850	—
DW test	0.746	0.243	1.097	1.080	—
*p* value^b^	*0.002*	*<0.001*	*0.028*	*0.024*	—

*Note:* *β*_0_: intercept; *β*_1_: linear regression slope coefficient (percentage points per year); *R* ^2^: coefficient of determination.

Abbreviations: CI, confidence interval; DW, Durbin–Watson statistic.

^a^ *p* value indicates the statistical significance of the linear trend slope (*β*_1_).

^b^ *p* value associated with the Durbin–Watson statistic, testing the null hypothesis of no first‐order serial autocorrelation (a significant result, *p *< 0.05, confirms the presence of serial autocorrelation in regression residuals, justifying the transition to generalized linear modeling in stage three).

**FIGURE 1:**
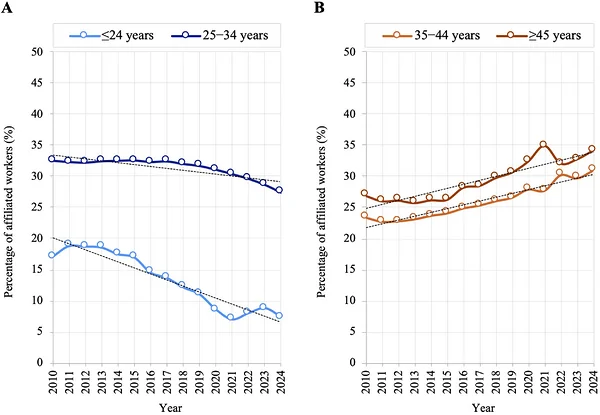
Temporal evolution and exploratory linear trends of the annual percentage distribution of affiliated construction workers in Ecuador, by age group (2010–2024). (A) Younger groups (≤24 and 25–34 years) displaying a continuous proportional contraction over time. (B) Older groups (35–44 and ≥45 years) exhibiting sustained demographic expansion. Dotted lines represent fitted simple linear regression trends.

### 3.2 Demographic Distribution and Exploratory Trend Analysis of TDOIs

Regarding the annual percentage distribution of TDOIs during the study period (Table 2; Figure 2), a pattern similar to that observed among affiliated workers was evident. Exploratory linear trend models explained a high proportion of temporal variance across all age groups (*R* ^2^ = 0.787–0.961; *p* < 0.001), and residual diagnostics confirmed significant first‐order serial autocorrelation in three of the models (DW = 0.613–1.052; *p* < 0.01). The 25–34 age bracket, however, satisfied the assumption of independent errors (DW = 1.796; *p* = 0.440). The proportion of TDOIs among workers aged ≤24 years declined from 35.53% to 4.53%, whereas those aged 25–34 years decreased from 38.83% to 25.79% (Figure 2A). In contrast, the proportion of TDOIs among workers aged 35–44 years increased from 16.31% to 30.31%, and those aged ≥45 years rose from 9.32% to 39.37% (Figure 2B).

**TABLE 2:** Annual percentage distribution of temporary disability occupational injuries (TDOIs) in the Ecuadorian construction sector by age group and exploratory linear trend analysis (2010–2024).

Year	Age group				Total, *N*
	≤24 (%)	25–34 (%)	35–44 (%)	≥45 (%)	
2010	35.53	38.83	16.31	9.32	515
2011	31.25	34.22	20.94	13.59	640
2012	30.40	35.76	19.35	14.49	987
2013	25.89	38.51	21.36	14.24	1545
2014	22.45	35.50	24.74	17.32	1617
2015	18.48	34.08	26.35	21.08	1385
2016	17.81	33.95	25.16	23.08	966
2017	13.77	34.21	25.59	26.43	719
2018	9.36	32.21	31.09	27.34	534
2019	11.36	31.03	30.83	26.77	493
2020	9.47	32.20	32.58	25.76	264
2021	6.87	29.55	33.43	30.15	335
2022	6.68	27.23	31.93	34.16	404
2023	5.88	27.45	28.63	38.04	510
2024	4.53	25.79	30.31	39.37	508
Intercept (*β_0_*)	4522.699	1676.052	−2089.332	−4009.924	
Slope (*β*_1_)	−2.234	−0.815	1.049	2.000	—
(95% CI)	(−2.562, −1.906)	(−1.010, −0.620)	(0.723, 1.375)	(1.758, 2.242)	—
*p* value^a^	*<0.001*	*<0.001*	*<0.001*	*<0.001*	—
Model fit (*R* ^2^)	0.943	0.864	0.787	0.961	—
DW test	0.613	1.796	0.959	1.052	—
*p* value^b^	*<0.001*	*0.440*	*0.008*	*0.008*	—

*Note:* *β*_0_: intercept; *β*_1_: linear regression slope coefficient (percentage points per year); *R* ^2^: coefficient of determination.

Abbreviations: CI, confidence interval; DW, Durbin–Watson statistic.

^a^ *p* value indicates the statistical significance of the linear trend slope (*β*_1_).

^b^ *p* value associated with the Durbin–Watson statistic, testing the null hypothesis of no first‐order serial autocorrelation (a significant result, *p *< 0.05, confirms the presence of serial autocorrelation in regression residuals, justifying the transition to generalized linear modeling in stage three).

**FIGURE 2:**
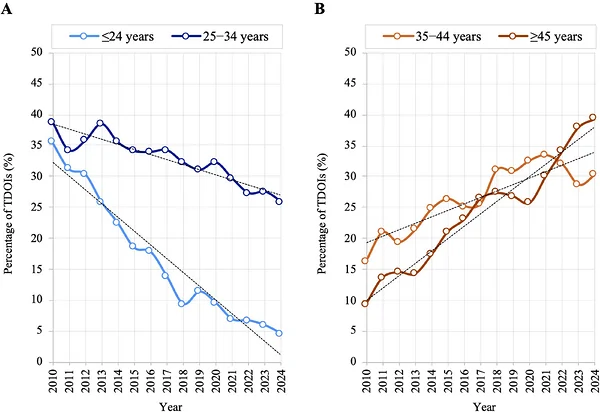
Temporal evolution and exploratory linear trends of the annual percentage distribution of TDOIs in the Ecuadorian construction sector, by age group (2010–2024). (A) Younger cohorts (≤24 and 25–34 years) exhibiting a sustained decline in their proportional share of total injuries. (B) Mature cohorts (35–44 and ≥45 years) showing a prominent upward trend, with workers aged ≥45 years becoming the primary injury group by 2024. Dotted lines represent fitted simple linear regression trends. TDOIs, temporary disability occupational injuries.

### 3.3 Long‐Term Age‐Specific Injury Rate Trends (GLM, EAPC) and COVID‐19 Sensitivity Analysis

Annual age‐specific injury IRs (per 1000 affiliated workers) and log‐linear generalized linear models (GLM) revealed divergent long‐term trajectories across age cohorts over the 15‐year study period (2010–2024) (Table 3; Figure 3).

**TABLE 3:** Annual age‐specific temporary disability occupational injuries (TDOI) incidence rates (per 1000 affiliated workers), log‐linear GLM trend parameters, and COVID‐19 sensitivity analysis in the Ecuadorian construction sector (2010–2024).

	Age group (years)			
Year	≤24	25–34	35–44	≥45
2010	16.57 (14.34, 19.15)	9.52 (8.29, 10.94)	5.56 (4.49, 6.89)	2.76 (2.08, 3.66)
2011	11.67 (10.16, 13.40)	7.44 (6.52, 8.51)	6.48 (5.47, 7.67)	3.66 (2.97, 4.52)
2012	13.47 (12.03, 15.09)	9.24 (8.32, 10.25)	7.06 (6.13, 8.14)	4.60 (3.91, 5.42)
2013	14.94 (13.54, 16.48)	12.75 (11.76, 13.81)	9.92 (8.91, 11.05)	5.92 (5.19, 6.76)
2014	13.83 (12.47, 15.32)	11.79 (10.87, 12.80)	11.29 (10.23, 12.45)	7.13 (6.34, 8.01)
2015	10.01 (8.85, 11.31)	9.64 (8.80, 10.55)	10.08 (9.09, 11.16)	7.38 (6.58, 8.27)
2016	10.00 (8.61, 11.61)	8.58 (7.70, 9.56)	8.26 (7.28, 9.37)	6.68 (5.86, 7.62)
2017	6.22 (5.11, 7.57)	6.52 (5.76, 7.39)	6.23 (5.39, 7.20)	5.72 (4.96, 6.59)
2018	3.77 (2.86, 4.98)	4.93 (4.24, 5.72)	5.84 (5.01, 6.81)	4.50 (3.82, 5.29)
2019	4.91 (3.78, 6.38)	4.67 (3.98, 5.47)	5.51 (4.70, 6.46)	4.16 (3.51, 4.94)
2020	3.73 (2.51, 5.52)	3.49 (2.82, 4.31)	3.93 (3.18, 4.85)	2.68 (2.11, 3.40)
2021	3.83 (2.55, 5.76)	3.78 (3.11, 4.61)	4.69 (3.89, 5.65)	3.37 (2.78, 4.09)
2022	3.81 (2.61, 5.57)	4.19 (3.47, 5.05)	4.81 (4.05, 5.72)	4.87 (4.11, 5.75)
2023	3.74 (2.61, 5.35)	5.33 (4.52, 6.29)	5.36 (4.56, 6.31)	6.49 (5.64, 7.47)
2024	3.70 (2.46, 5.57)	5.65 (4.76, 6.70)	5.87 (5.02, 6.88)	6.94 (6.05, 7.98)
*Full series (2010–2024)*				
Model distribution	Poisson GLM	Negative binomial GLM	Negative binomial GLM	Negative binomial GLM
Dispersion (deviance/df)	0.530	1.341	1.637	1.258
Slope (*β*_1_)	−0.105	−0.046	−0.020	0.041
EAPC (95% CI)	−10.00 (−11.48, −8.48)	−4.50 (−5.92, −3.07)	−2.00 (−3.72, −0.25)	4.20 (2.53, 5.89)
*Sensitivity analysis (Excl. 2020–2021)*				
Slope (*β*_1_)	−0.108	−0.044	−0.015	0.048
EAPC (95% CI)	−10.25 (−11.80, −8.68)	−4.30 (−5.81, −2.76)	−1.50 (−3.30, 0.33)	4.90 (3.15, 6.68)

*Note:* IR: incidence rate per 1000 affiliated construction workers; *β*_1_: log‐linear regression slope coefficient (ln(rate) per year); EAPC: estimated annual percentage change [EAPC = (exp(*β*_1_) − 1) × 100]. Annual rates are presented as point estimates with exact 95% Poisson confidence intervals. Full series covers the complete 15‐year period (2010–2024; *n* = 15 years); sensitivity analysis excludes the COVID‐19 pandemic disruption period (2020–2021; *n* = 13 years). Log‐linear generalized linear models (GLM) were fitted with log‐transformed annual workforce as an offset (ln(workforce)).

Abbreviation: CI, confidence interval; GLM, generalized linear model.

**FIGURE 3:**
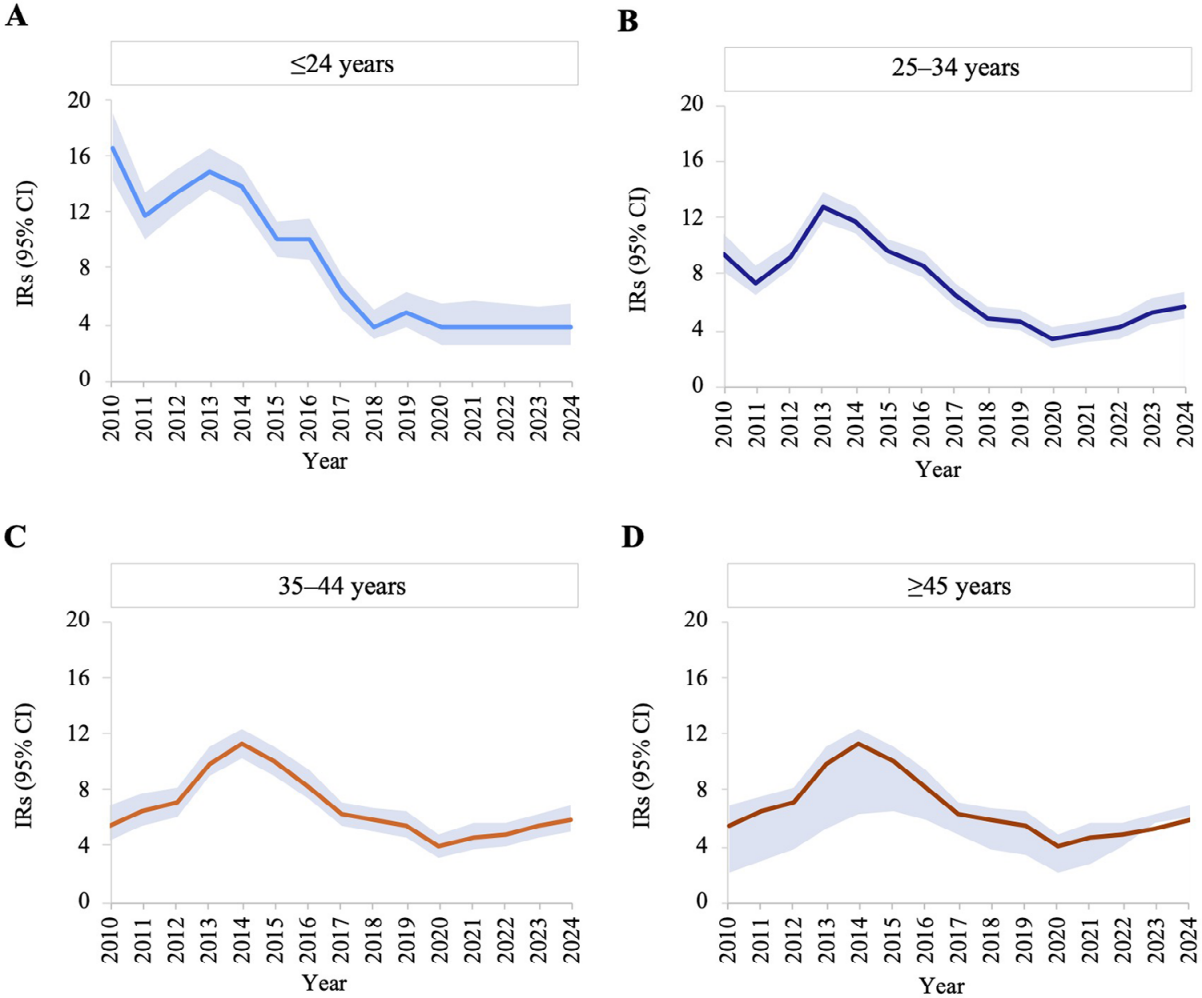
Annual age‐specific TDOI incidence rates (per 1000 affiliated construction workers) with 95% confidence intervals in Ecuador (2010–2024). (A) Workers aged ≤24 years exhibiting a sharp, sustained long‐term decline in injury rate. (B) Workers aged 25–34 years showing a moderate downward trend following a peak around 2013–2014. (C) Workers aged 35–44 years displaying a gradual overall decline after reaching peak rates in 2014. (D) Workers aged ≥45 years demonstrating a sustained upward long‐term trend, escalating to become the cohort with the highest incidence rate by 2024. Solid lines represent annual point estimates; shaded areas denote exact 95% Poisson confidence intervals. CI: confidence interval; IRs, incidence rates.

Over the full series (2010–2024), statistically significant downward trends in occupational injury risk were documented among younger and middle‐aged cohorts (Figure 3A–C). The most pronounced relative reduction occurred in the youngest group (≤24 years), whose IR dropped steeply from 16.57 per 1000 workers (95% CI: 14.34–19.15) in 2010 to 3.70 (95% CI: 2.46–5.57) in 2024 (Figure 3A), yielding an EAPC of −10.00% (*β*_1_ = −0.105; 95% CI: −11.48% to −8.48%; *p* < 0.001). Significant annual rate decreases were also recorded for workers aged 25–34 years (EAPC = −4.50%; 95% CI: −5.92% to −3.07%; *p* < 0.001) (Figure 3B) and 35–44 years (EAPC = −2.00%; 95% CI: −3.72% to −0.25%; *p* = 0.025) (Figure 3C).

In stark contrast, workers aged ≥45 years exhibited a statistically significant upward long‐term trend in injury risk (Figure 3D). The IR for this older cohort increased more than 2.5‐fold, rising from 2.76 per 1000 workers (95% CI: 2.08–3.66) in 2010 to 6.94 (95% CI: 6.05–7.98) in 2024, corresponding to an EAPC of +4.20% (*β*_1_ = 0.041; 95% CI: 2.53%–5.89%; *p* < 0.001).

The unified interaction GLM confirmed a highly significant differential temporal trajectory across age groups (Year × Age Group interaction likelihood‐ratio test, *p* < 0.001). Relative to the youngest cohort (≤24 years), the annual rate change coefficient showed significant slope heterogeneity for workers aged 25–34 years (*β *= 0.059, *p* < 0.001), 35–44 years (*β *= 0.085, *p* < 0.001), and ≥45 years (*β *= 0.146, *p* < 0.001). This formal interaction test confirms that the upward incidence trend observed in older workers (≥45 years) diverged significantly from the steep downward trajectory of the youngest group, statistically validating the central hypothesis of workforce risk shift and inversion.

The COVID‐19 sensitivity analysis, which excluded the pandemic period (2020–2021), confirmed high parameter stability and slope consistency across the primary risk cohorts (≤24 and ≥45 years) (Table 3). The direction and statistical significance of the EAPCs remained highly robust for the youngest group (EAPC = −10.25%; 95% CI: −11.80% to −8.68%) and the older cohort (EAPC = +4.90%; 95% CI: 3.15%–6.68%). For the 25–34 age group, the downward trend also remained statistically significant (EAPC = −4.30%; 95% CI: −5.81% to −2.76%). However, for the mid‐career cohort (35–44 years), whereas the trajectory remained negative (EAPC = −1.50%), statistical significance was no longer retained when excluding 2020–2021 (95% CI: −3.30% to 0.33%), indicating that the slight long‐term downward trend in this specific subgroup is sensitive to pandemic‐era rate dips.

## 4 Discussion

The demographic analysis of workers affiliated with the SGRT in Ecuador's construction sector reveals a progressive shift toward older age cohorts. This aging trend is partly driven by the country's broader demographic transition, characterized by a sustained decline in fertility rates over the past two decades [30]. However, as this study exclusively accounts for salaried workers within the formal social security system, it is important to situate these findings within the broader context of labor market dynamics in Latin America and the Caribbean. Youth employment in the region is characterized by high levels of informality, particularly in sectors like construction. Recent literature emphasizes the structural challenges young people face, such as precarious subcontracting and frequent turnover, which undermine stable employment and limit access to social security coverage [31]. This situation is further reflected in regional employment outlooks, which highlight the persistence of informal hiring practices and their implications for youth employment in the region [32]. Focusing on Ecuador, local studies identify informality and temporary contracts as major determinants of youth unemployment, underscoring the limited reach of social security among young workers [33].

Furthermore, the gradual decline in new young entrants may be exacerbated by the industry's high physical demands and inherent occupational risks, which, coupled with less competitive salaries compared to other sectors, discourage generational replacement [21, 34]. This lack of new recruits leads to a stagnant workforce where older age groups become increasingly overrepresented, a phenomenon consistent with international observations of aging workforces in the construction industry [10, 35, 36].

The drastic reduction in injury rates among workers aged ≤24 years warrants a cautious interpretation. Rather than reflecting a definitive improvement in safety standards, this trend may be driven by significant underreporting, a phenomenon widely documented in young populations with high job insecurity [37]. Young workers, often in precarious or temporary positions, may be reluctant to report minor injuries to avoid potential job loss, a situation frequently compounded by a lack of awareness regarding their rights within the SGRT framework [32]. Conversely, the sustained increase in IRs among the ≥45 age group indicates an evolving pattern of occupational vulnerability that aligns with demographic and workplace dynamics. Although our ecological study design precludes establishing direct individual‐level causation, this population‐level trend is consistent with plausible mechanisms documented in occupational health literature, such as accumulated biomechanical exposure and age‐related physiological shifts [38, 39]. In physically demanding sectors like construction, theoretical models suggest a potential mismatch between age‐associated variations in physical functional capacity (e.g., balance, muscle strength, and sensory acuity) and unadapted job‐site demands [40]. These observations align with findings from other industrial settings, where older cohorts frequently exhibit heightened vulnerability to workplace injuries [7].

The epidemiological shift documented in this study has direct implications for public health policy, occupational risk prevention, and labor legislation in Ecuador and the broader Latin American region. Historically, occupational safety regulations in developing countries have followed a “one‐size‐fits‐all” approach, focusing primarily on acute safety hazards and assuming a younger, physically resilient workforce. However, the observed risk inversion highlights the urgent need to transition toward age‐sensitive occupational health policies. First, at the enterprise and sector levels, managing an aging workforce requires the integration of proactive ergonomic interventions, dynamic risk assessments, and workload adjustments tailored to older workers. Strategies such as rotating physical tasks, enforcing mandatory rest intervals, setting biomechanical load limits, and implementing functional capacity evaluations can help mitigate cumulative physical stress and prevent debilitating injuries among workers aged 45 years and older. Additionally, comprehensive health promotion programs targeting chronic musculoskeletal and metabolic conditions should be incorporated into institutional occupational safety agendas. Second, from a regulatory and legislative perspective, national labor protection frameworks in Latin America, including Ecuador's General Insurance for Work Risks (SGRT), must evolve to address changing demographic realities. Current technical standards lack specific guidelines for age‐structured risk management in heavy industries. Policymakers should update national occupational safety protocols to mandate age‐specific preventative measures and continuous health surveillance. Finally, addressing the high rate of informal employment in the construction sector, estimated at 70%, remains a major public health challenge. Incentive mechanisms for formal social security affiliation are essential to extend occupational health coverage, injury reporting, and preventive monitoring to the most vulnerable segments of the workforce.

### 4.1 Strengths and Limitations

This study presents notable strengths, including a 15‐year national longitudinal design (2010–2024) utilizing official administrative records from the SGRT to evaluate population‐level epidemiological shifts across age groups in the Ecuadorian construction sector. However, several methodological limitations inherent to secondary administrative data must be acknowledged when interpreting these findings.

First, regarding secondary data limitations, the analysis relies exclusively on aggregated administrative registries from the IESS. Consequently, the research team had no control over primary data collection protocols, variable definition, or initial classification criteria. Second, underreporting remains a structural issue affecting occupational health statistics across all age groups. Younger workers may underreport injuries due to job insecurity or temporary contracting, whereas older cohorts might normalize risks or fear forced retirement; thus, the IRs reported here represent conservative estimates of the actual sectoral injury burden.

Third, the dataset reflects partial workforce coverage, as it includes only formally affiliated, salaried construction workers. Given that the Ecuadorian construction industry operates with an estimated structural informality rate of approximately 70% [20], the external validity of these results is constrained, and findings cannot be directly generalized to informal workers who lack social security protection. Fourth, the database suffers from an absence of clinical variables, lacking information on preexisting comorbidities, functional capacity metrics, and lifestyle or health behaviors that could provide deeper insight into individual physical vulnerability.

Fifth, there is a lack of injury severity assessment, as the administrative records categorize injuries solely as TDOIs without detailing anatomical injury sites, specific diagnostic classifications, days lost, or standardized severity scores. Sixth, because analyses were performed on aggregated demographic cohorts over time rather than tracking individuals’ longitudinal trajectories, the findings are subject to potential ecological bias. Seventh, due to the observational and descriptive nature of the study design, causal inference is limited; the observed temporal trends demonstrate robust epidemiological associations rather than direct causal pathways between biological aging, workplace exposures, and injury occurrence. Finally, although log‐linear GLMs effectively model overall long‐term epidemiological trends via EAPCs, this approach summarizes the average 15‐year change and may smooth out short‐term non‐linear fluctuations. Furthermore, sensitivity analyses revealed that while primary risk cohorts (≤24 and ≥45 years) displayed robust parameter stability, mid‐career workers (35–44 years) showed marginal trend shifts where statistical significance depended on the inclusion of pandemic‐period data.

## 5 Conclusions

Between 2010 and 2024, substantial demographic ageing of the SGRT‐affiliated construction workforce occurred alongside divergent age‐specific occupational injury trends. Prior to implementing new occupational safety and health policies, it is imperative to consolidate a research foundation that elucidates the underlying causes of the diverging injury trends between younger cohorts and older workers.

Future research should further examine the ongoing generational shift within the Ecuadorian construction workforce and its broader implications for occupational safety and sectoral sustainability. Workers aged ≥45 years are largely drawn from Generation X, a cohort shaped by different educational, training, and labor market conditions than younger generations. In contrast, younger cohorts, particularly millennials, may display different occupational expectations and educational trajectories, which could influence their participation in physically demanding sectors such as construction. Future studies should therefore consider generational cohort classification to better understand how age, training background, and work expectations interact in shaping occupational risk patterns and workplace dynamics. In addition, research is needed to explore how the incorporation of new technologies may affect both safety conditions and workforce composition. From a broader macroeconomic perspective, future analyses should also compare temporal changes in injury IRs with fluctuations in construction activity and with the sector's contribution to Ecuador's gross domestic product over the same period.

## Author Contributions

**Antonio Ramon Gómez‐García**: conceptualization, investigation, funding acquisition, writing – original draft, writing – review and editing, validation, methodology, supervision. **Cesar Eduardo Espinoza Samaniego**: data curation, formal analysis, visualization, conceptualization, investigation, funding acquisition, writing – original draft.

## Funding

This study was funded by Universidad Espíritu Santo under the research project (code: 2024‐POST‐001): monitoring the employment, labor, and health conditions of older workers in Ecuador: analysis and recommendations for healthier aging.

## Ethics Statement

The authors have nothing to report.

## Conflicts of Interest

The authors declare no conflicts of interest.

## Data Availability

The data that support the findings of this study are available in Instituto Ecuatoriano de Seguridad Social at https://www.iess.gob.ec/es/estadisticas, reference number Boletines Estadísticos 2010–2024. These data were derived from the following resources available in the public domain: Instituto Ecuatoriano de Seguridad Social, https://www.iess.gob.ec/es/estadisticas.
